# Multitarget Tracking Algorithm Based on Adaptive Network Graph Segmentation in the Presence of Measurement Origin Uncertainty

**DOI:** 10.3390/s18113791

**Published:** 2018-11-06

**Authors:** Tianli Ma, Song Gao, Chaobo Chen, Xiaoru Song

**Affiliations:** 1 Autonomous Systems and Intelligent Control International Joint Research Center, Xi’An Technological University, Xi’an 710021, China; gaos@xatu.edu.cn (S.G.); chenchaobo@xatu.edu.cn (C.C.); songxiaoru@xatu.edu.cn (X.S.); 2 School of Mechatronic Engineering, Xi’An Technological University, Xi’an 710021, China

**Keywords:** network flow theory, multitarget tracking, spectral clustering, A* search algorithm, RTS smoother, integer programming

## Abstract

To deal with the problem of multitarget tracking with measurement origin uncertainty, the paper presents a multitarget tracking algorithm based on Adaptive Network Graph Segmentation (ANGS). The multitarget tracking is firstly formulated as an Integer Programming problem for finding the maximum a posterior probability in a cost flow network. Then, a network structure is partitioned using an Adaptive Spectral Clustering algorithm based on the Nyström Method. In order to obtain the global optimal solution, the parallel A* search algorithm is used to process each sub-network. Moreover, the trajectory set is extracted by the Track Mosaic technique and Rauch–Tung–Striebel (RTS) smoother. Finally, the simulation results achieved for different clutter intensity indicate that the proposed algorithm has better tracking accuracy and robustness compared with the A* search algorithm, the successive shortest-path (SSP) algorithm and the shortest path faster (SPFA) algorithm.

## 1. Introduction

The purpose of multitarget tracking is to jointly estimate the number of targets and their state of motion from sensor data [1]. During the past decade, it has developed in a variety of directions, such as Air-Traffic Control [2], Marine Monitoring [3], Computer Vision [4], Autonomous Vehicle and Robot [5], etc. At present, multitarget tracking has achieved substantial advances [6]. However, the measurement origin uncertainty, for instance the unknown and time-varying number of targets, clutters, jamming signals and so forth seriously deteriorates the performance of the multitarget tracking system. Resolving the uncertainty of the measurement origin is a computationally expensive task which relied on the prior information about the target motion. To find the mapping from each measurement to its origin is often called measurement-to-track association or just data association [7,8].

Markov Chain Monte Carlo Data Association algorithm (MCMCDA) based on Bayesian Inference [9] and the Probability Hypothesis Density filter (PHD) based on finite set statistics (FISST) [10] have been proposed to cope with this problem of tracking multiple targets with measurement uncertainty. The MCMCDA algorithm can be viewed as a deferred-logic method since a track is decided based on the current and past measurements. It uses the Markov Chain Monte Carlo sampling instead of enumerating over all possible associations. In a PHD filter, it can estimate the target states by recursively computing the first-order moment of the multi-target state posterior probability distribution, without using the complex data association techniques. However, it was not designed to estimate the trajectories of targets. For this problem, Ba-Ngu Vo [11] proposed a newly labeled Random Finite Set (RFS) approach, known as the generalized labeled multi-Bernoulli (GLMB) filter, it can output trajectories and has a better performance in harsh environments.

Bar-Shalom [12] proposed an multidimensional assignment algorithm for solving the data association problem. In essence, the data association problem is converted to a combinatorial optimization problem under certain linear constraints where the total distance/benefit of assigning targets to measurements is minimized/maximized. There is a wide range of algorithms, such as Greedy algorithm [13], Genetic algorithm [14] and Lagrange relaxation theory [15], are used to find the sub-optimum solutions of the multidimensional assignment problem [16]. These approaches, while effective, need to solve the Non-deterministic Polynomial Complete (NPC) with a large amount of data. Goldberg [17] constructed an efficient min-cost flow framework. It has applied a scaling push relabel method to find the optimal solution. Under this framework, Zhang [18] formulated the multitarget data association problem as a maximum a posteriori (MAP) problem. It is mapped into the cost flow network and finds the global optimum solution by depending on the min-cost flow algorithm. An approach combining Dynamic Programming (DP) and Successive Shortest-Path algorithm (SSP) is presented by using Hidden Markov Model (HMM) in [19]. The multitarget tracking problem is formulated as an Integer Linear Programming (ILP) problem, and a greedy, successive shortest-path algorithm is used to reduce the runtime costs. For k=1, this algorithm can obtain the global optimal solution. For k>1, it only obtains the approximate solutions, where *k* is the unknown number of targets. The Shortest Path Faster algorithm (SPFA) is used to solve the Integer Programming problem of the min-cost flow network and quickly obtains the global optimal solution in [20]. The algorithm improves the robustness and tracking accuracy. In [21], an A* based tracking association algorithm is presented. The integer assumption is relaxed to the standard Linear Programming (LP) problem so that the global optimal solution can be obtained by the A* search algorithm.

The above-mentioned approaches are mainly focused on the object tracking based on video image. The available information of image targets are more than that of point targets. In addition, less clutter leads to simple network structure so that the aforementioned algorithms have a good tracking performance. For the problem of multiple point targets tracking in the presence of measurement origin uncertainty, the network may become more complicated that result in an enormous computational burden.

In this paper, a multitarget tracking algorithm based on adaptive network graph segmentation is proposed to address the problem of tracking multitarget with measurement uncertainty. Parallel search strategy is employed to solve the NP-complete problem. The network flow model of multitarget tracking is divided into different sub-graphs. The optimal trajectory is extracted by using the A* search algorithm. Our main contributions are: (1) a parallel network search framework is presented to cope with the multitarget tracking in the presence of measurement origin uncertainty; and (2) we proposed a adaptive spectral clustering algorithm based on the Nyström Method to obtain the network segmentation results for an unknown cluster number data set.

The rest of paper is organized as follows: the problem of multiple targets tracking is formulated as a cost flow network, and transform it into an Integer Programming problem in Section 2. In Section 3, the A* search algorithm is briefly reviewed. The original contribution of the paper is presented in Section 4, where we describe the adaptive spectral clustering algorithm based on the Nyström Method. Simulation results are provided in Section 5. Conclusions and possible extensions appear in Section 6.

## 2. Problem Formulation

The multitarget data association problem is regarded as a cost flow network. Let $Z=\{z_{i}\}$ be a set of measurements, $z_{i}=\left\{\phi_{i},\varphi_{i},t_{i}\right\}$, where $\phi_{i},\varphi_{i}$ is the position in *x* and *y*-axes, respectively. $t_{i}$ is the time step of the measurement $z_{i}$. $T_{k}=\left\{z_{k_{1}},z_{k_{2}},\ldots z_{k_{n}}\right\}$ represents a trajectory. The set of trajectories is $T=\{T_{1},T_{2},\ldots ,T_{L}\}$, and the number of trajectories *L* is unknown. The key of the data association is to compute the maximum a posteriori (MAP) estimate of T given the measurement set Z:

(1) $$\begin{array}{cc} T^{*} & =\underset{T}{\arg\max}\;P\left(T|Z\right)\\ & =\underset{T}{\arg\max}\;P\left(Z|T\right)P\left(T\right)\\ & =\underset{T}{\arg\max}\;\prod_{i}P(z_{i}|T)P\left(T\right). \end{array}$$

Assume that tracks are independent from each other. The cost flow network framework of multitarget tracking is as follows:

(2) $$P\left(T\right)=\prod_{T\in T}P(T),$$

(3) $$P\left(T\right)=P_{s}\left(z_{k_{0}}\right)P_{l}\left(z_{k_{1}}|z_{k_{0}}\right)\ldots P_{l}\left(z_{k_{n}}|z_{k_{n-1}}\right)P_{t}\left(z_{k_{n}}\right),$$

where P(T) is modeled as a Markov Chain. $P_{s}\left(z_{k_{0}}\right)$ is the initialization probability of a track starting at $z_{k_{0}}$, $P_{t}\left(z_{k_{n}}\right)$ is the termination probability of a track ending at $z_{k_{n}}$, and $P_{l}\left(z_{k_{j}}|z_{k_{i}}\right)$ is the transition density from measurement $z_{k_{i}}$ to measurement $z_{k_{j}}$. $P(z_{i}|T)$ denotes the likelihood function of measurement $z_{i}$, which represents a measurement being a true target or a false alarm. In this paper, all measurements are regarded as targets; this means that $P(z_{i}|T)=1$, and the posterior probability of trajectory set T is calculated as follows:

(4) $$T^{*}=\underset{T}{\arg\max}\;\prod_{T\in T}P(T).$$

To take advantage of the concept of network flow in Network Optimization [22], the indictor variable $f_{i,j}$ is defined as the directed flow variable that from measurement $z_{i}$ to measurement $z_{j}$. $f_{s,i}$ and $f_{i,t}$ represent the starting flow variable and terminated flow variable, respectively. Depending on the flow conversation method [22], for all nodes $z_{i}$, the sum of flows arriving at node $z_{i}$ is equal to the sum of outgoing flows from node $z_{i}$. For any track $T_{k}$, it is satisfies the following equation:

(5) $$f_{s,i}+\sum_{j}f_{j,i}=\sum_{i}f_{i,j}+f_{i,t}.$$

Moreover, the cost flow network must guarantee that only one node is represented by a target in a moment. Let the upper bound of the sum of outgoing flows from node $z_{i}$ is 1. For any node, the constraint is

(6) $$\forall z_{i},z_{j}\;\;\;\;\sum f_{i,j}\leq 1.$$

**Figure 1:**
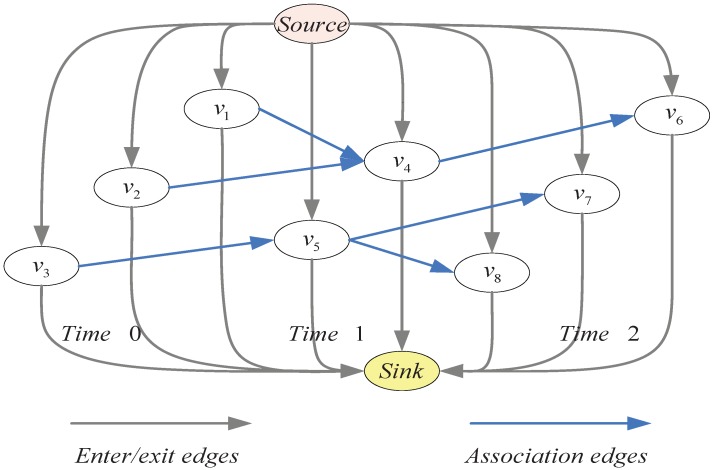
An example of cost flow model with three time steps.

Taking into account that the target may appear or disappear from any location in the cost flow network, the source and sink node are introduced in [18], which is connected to each node, respectively. We transform the Network Optimization problem of Equation (4) into the IP problem, and the logarithm of the objective function can be rewritten as

(7) $$\begin{array}{cc} T^{*} & =\underset{T}{\arg\min}\;\sum_{T_{k}\in T}-\log P(T_{k})\\ & =\underset{T}{\arg\min}\;\sum_{T_{k}\in T}\left(c_{s,i}f_{s,i}+\sum_{j}c_{i,j}f_{i,j}+c_{j,t}f_{j,t}\right)\\ & =\underset{T}{\arg\min}\;\sum_{i}c_{s,i}f_{s,i}+\sum_{i,j}c_{i,j}f_{i,j}+\sum_{j}c_{j,t}f_{j,t}, \end{array}$$

where $c_{s,i}$ is the cost of the flow from the source node to measurement $z_{i}$, $c_{i,j}$ is the cost of the flow from measurement $z_{i}$ to measurement $z_{j}$, and $c_{j,t}$ is the cost of the flow from measurement $z_{j}$ to the sink node. Figure 1 shows an example of the cost flow network. The IP problem of multitarget tracking with measurement uncertainty can be described as

(8) $$\begin{array}{c} \min\;\sum_{i}c_{s,i}f_{s,i}+\sum_{i,j}c_{i,j}f_{i,j}+\sum_{j}c_{j,t}f_{j,t} \end{array}$$

(9) $$\begin{array}{c} \begin{array}{ccc} s.t. & \forall z_{i},z_{j}, & \sum f_{i,j}\leq 1\\ & \forall z_{i},z_{j}, & f_{i,j}\geq 0. \end{array} \end{array}$$

The cost can be defined as follows:

(10) $$\left\{\begin{array}{cc} c_{s,i} & =-\log P_{s}\left(z_{i}\right),\\ c_{i,j} & =-\log P_{l}\left(z_{j}|z_{i}\right),\\ c_{j,t} & =-\log P_{t}\left(z_{j}\right). \end{array}\right.$$

For the IP problem of multitarget tracking, the traditional algorithms have a relatively higher computational cost due to the large number of network nodes. Hence, a parallel processing technology based on the A* search algorithm is presented to find the optimal solution.

## 3. Description of the A* Search Algorithm

The A* search algorithm is a heuristic graph search method which guides the search process by using the characteristic information of the problem. For the min-cost flow problem, the A* algorithm searches from the origin node, calculates and estimates the cost of each extended node, chooses the extended node that has a minimum cost and stops it when the algorithm reaches the destination node. Assuming that an evaluation function $f^{*}\left(x\right)$ is designed to estimate the minimum cost from origin node $s_{o}$ through node *x* to destination node $s_{d}$. The estimated minimum cost is calculated as follows:

(11) $$f^{*}\left(x\right)=g^{*}\left(x\right)+h^{*}\left(x\right),$$

where $g^{*}\left(x\right)$ is the cost from origin node $s_{o}$ to the node *x*, $h^{*}\left(x\right)$ is the lower bound on the minimum cost from node *x* to destination node $s_{d}$, and h(x) is the actual value from the node *x* to destination node $s_{d}$, $h^{*}\left(x\right)\leq h\left(x\right)$. In order to ensure the optimality of the algorithm, Admissibility and Consistency conditions must be satisfied [23]. Admissibility condition: $f^{*}\left(x\right)$ never overestimates the true cost of a solution along the current path through. Consistency condition: A heuristic function h(x) is consistent if, for every node *x* and every successor $x^{\prime }$ of *x*, the estimated cost of reaching the goal from *x* is no greater than that of the step cost of getting to $x^{\prime }$ form *x* plus the estimated cost of reaching the goal from $x^{\prime }$

(12) $$h^{*}\left(x\right)+e\left(x,x^{\prime }\right)\leq h^{*}\left(x^{\prime }\right).$$

In the implementation of the A* search algorithm, two lists need to be built, named Open list and Close list. Open list is the set of nodes that have been calculated, and that are candidates for the selection of the next node. Close list is the set of nodes that have been selected, and that are not in Open list. In the initial stage, Open list contains a source node and Close list is empty. During the iterative process, the A* search algorithm calculates the evaluation function of each node in Open list chooses the node with the minimum cost and judges whether it is the termination node. If so, the algorithm is done. Otherwise, it extends all adjacent nodes and calculates the cost function of each node. If the solution exists, the A* search algorithm can guarantee obtaining the optimal solution [24].

The A* search algorithm can be expressed as follows: here, the Open list and Close list are denoted as *O* and *C*. *E* is the set of edges:

Step 1 Initialization:
  Set $x_{i}=x_{s}$, $f^{*}\left(x_{i}\right)=0$; $f^{*}\left(x_{j}\right)=\infty$, $g^{*}\left(x_{j}\right)=\infty$, $\forall x_{j}\neq x_{i}$; $O=\left\{x_{i}\right\}$, C=O.
Step 2 Node Selection:
  Choose $x_{i}\in Arg\min_{x_{j}\in O}f^{*}\left(x_{j}\right)$, $C=C⋃\{x_{i}\}$, $O=O\backslash \{x_{i}\}$.
Step 3 Stop Rule:
  If $x_{i}=x_{d}$, then stop. otherwise, continue.
Step 4 Update $f^{*}\left(x_{j}\right)$ and $g^{*}\left(x_{j}\right)$:
  For each $x_{j}\in E\left(x_{i}\right)$: If $g^{*}\left(x_{i}\right)+e(x_{i},x_{j})+h^{*}\left(x_{j}\right)<f^{*}\left(x_{j}\right)$, then $g^{*}\left(x_{j}\right)=g^{*}\left(x_{i}\right)+e(x_{i},x_{j})$;
  $f^{*}\left(x_{j}\right)=g^{*}\left(x_{i}\right)+e(x_{i},x_{j})+h^{*}\left(x_{j}\right)$.
  If $x_{j}\notin O$, $O=O⋃\{x_{j}\}$. Go back to Step 2.

## 4. Multitarget Tracking Algorithm Based on Adaptive Network Graph Segmentation

**Figure 2:**
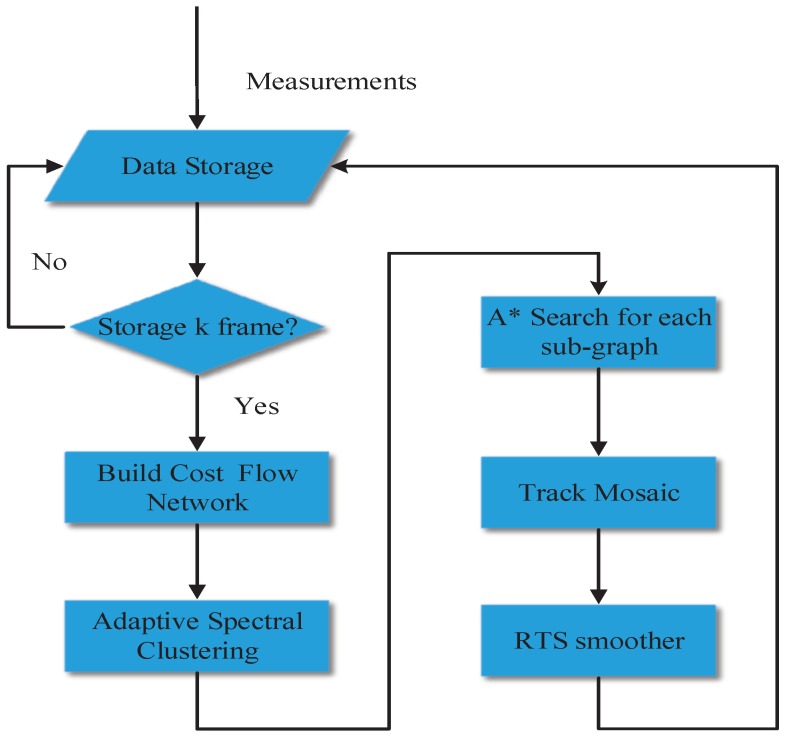
The flow chart of multitarget tracking algorithm based on adaptive network graph segmentation.

Under the multitarget tracking environment, the A* search algorithm have two obvious defects that long running time and large storage space. To solve the problem, an adaptive spectral clustering algorithm is presented to segment the cost flow network, and the A* search algorithm is used to find the optimal track of segmented sub-graph. To take advantage of track mosaic technology, the optimal track of each sub-graph is combined, and the combined track is smoothed by the Rauch–Tung–Striebel smoother. The flow chart of the proposed algorithm is depicted in Figure 2.

### 4.1. Adaptive Spectral Clustering

The segmentation problem of graph structure represented by the dissimilarity degree between nodes is the combinatorial optimization problem, which is NP-hard. The general solution is to consider the continuous relaxation form for this problem. Spectral clustering is an unsupervised learning method based on graph theory [25] for arbitrary image shapes. It uses the eigenvalue decomposition of graph matrix to build a spectral mapping space of the original data set, and the new space is partitioned by the K-means algorithm.

Let $G_{1}\left(V,E,W\right)$ be an undirected graph that transforms from the directed graph $G_{0}\left(V_{0},E_{0}\right)$ with vertex set $V=(v_{1},v_{2},\ldots ,v_{N})$ and edge set $E={\left(e_{ij}\right)}_{i,j=1,2,\ldots ,m}$. We assume that the edge-weighted adjacency matrix of undirected graph $W={\left(w_{ij}\right)}_{i,j=1,2,\ldots ,m}$, which is also called affinity matrix, is nonnegative, $w_{ij}=w_{ji}\geq 0$. If $w_{ij}=0$ that means the node $v_{i}$ and $v_{j}$ are not connected. Here, the weight of two nodes $w_{ij}$ is the cost value $c_{ij}$ in the directed graph model $G_{0}\left(V_{0},E_{0}\right)$. The sets $\Psi_{1}....\Psi_{k}$ are the subsets of the graph. For sets $\Psi_{1}....\Psi_{k}$, $\Psi_{1}⋃\Psi_{2}..⋃\Psi_{k}=V$ and $\Psi_{i}⋂\Psi_{j}=Ø,i\neq j$. Let $cut(\Psi_{1},\Psi_{2},\ldots ,\Psi_{k})$ be the sum of the cuts between sets $\Psi_{1},\Psi_{2},\ldots ,\Psi_{k}$:

(13) $$cut\left(\Psi_{1},\Psi_{2},\ldots ,\Psi_{k}\right)=\sum_{i=1}^{k}cut(\Psi_{i},{\bar{\Psi }}_{i}),$$

where ${\bar{\Psi }}_{i}$ denotes the complement $V\backslash \Psi_{i}$. The purpose of the spectral clustering is to find the sets $\Psi_{1},\Psi_{2},\ldots ,\Psi_{k}$ such that MNcut is minimized. The objective function is given as follows:

(14) $$MNcut=\sum_{i=1}^{n}\frac{cut(\Psi_{i},{\bar{\Psi }}_{i})}{\sum_{u\in \Psi_{i},v\in {\bar{\Psi }}_{i}}w_{u,v}}.$$

In [26], eigenvectors are clustered in the subspace that is generated by the first *k* eigenvectors of normalized Laplacian matrix. The degree matrix of the graph D and the normalized Laplacian matrix $L_{sym}$ are defined as

(15) $$D=\left\{\begin{array}{c} D_{ii}=\sum_{j}w_{ij},\\ D_{ij}=0\;i\neq j, \end{array}\right.$$

(16) $$L_{sym}=I-D^{-1/2}WD^{-1/2}.$$

Unfortunately, W grows as the square of the number of elements in the grouping problem, and it quickly becomes infeasible to fit W in memory. Hence, an adaptive spectral clustering based on the Nyström Method is proposed to reduce the complexity of the time and space. The Nyström Method is a technique for finding numerical approximations to eigenfunction problems.

Assuming that randomly sample *n* points from vertex set V and the number of the remaining points is N−n. Now, partition the affinity matrix W as

(17) $$W=\left[\begin{array}{cc} A & B\\ B^{T} & C \end{array}\right],$$

where $A\in R^{n\times n}$ represents the sub-block of weights among the random samples, $B\in R^{(N-n)\times n}$ contains the weights between the sample points and the rest of points, and $C\in R^{\left(N-n)\times (N-n\right)}$ denotes the weights matrix between all of the remaining points. Let $\bar{U}$ represent the approximate eigenvectors of W:

(18) $$\bar{U}=\left[\begin{array}{c} U\\ B^{T}U\Lambda^{-1} \end{array}\right].$$

The approximation of W, which we denote $\hat{W}$, can be written as

(19) $$\begin{array}{cc} \hat{W} & =\bar{U}\Lambda {\bar{U}}^{T}\\ & =\left[\begin{array}{c} U\\ B^{T}U\Lambda^{-1} \end{array}\right]\Lambda \left[\begin{array}{cc} U^{T} & \Lambda^{-1}U^{T}B \end{array}\right]\\ & =\left[\begin{array}{cc} A & B\\ B^{T} & B^{T}A^{-1}B \end{array}\right]. \end{array}$$

From Equations (17) and (19), C is approximated by $B^{T}A^{-1}B$. Therefore, calculating the affinity matrix between remaining points is avoided. It is noteworthy that the columns of $\bar{U}$ are not orthogonal. We need to orthogonalize $\bar{U}$. If A is a positive definite matrix, $A^{-1/2}$ represents the symmetric positive definite square root of A. Let $Q=A+A^{-1/2}BB^{T}A^{-1/2}$, and diagonalize Q as $Q=U_{s}\Lambda_{s}U_{s}^{T}$. If the matrix $U_{v}$ is defined as

(20) $$U_{v}=\left[\begin{array}{c} A\\ B^{T} \end{array}\right]A^{-1/2}U_{s}\Lambda_{s}^{-1/2},$$

then the affinity matrix *W* is diagonalized by $U_{v}$ and $\Lambda_{s}$. Without calculating $B^{T}A^{-1}B$, a simple approach is proposed to calculated the row sums of $\hat{W}$:

(21) $$\hat{d}=\left[\begin{array}{cc} a_{r} & b_{r}\\ b_{c} & B^{T}A^{-1}b_{r} \end{array}\right],$$

where $a_{r},b_{r}\in R^{N-n}$ denote the rows sum of A and B. $b_{c}\in R^{n}$ denotes column sum of B. The normalized A and B can be obtained by $\hat{d}$. The elements of the normalized A and B are given by

(22) $$A_{ij}=\frac{A_{ij}}{\sqrt{{\hat{d}}_{i}{\hat{d}}_{j}}},i,j=1,\ldots ,n,$$

(23) $$B_{ij}=\frac{B_{ij}}{\sqrt{{\hat{d}}_{i}{\hat{d}}_{j+N}}},i=1,\ldots ,n,j=1,\ldots ,N-n.$$

The number of clusters is generally determined by human experience and background knowledge. Next, the relationship between the spectrum of the weight matrix and the number of clusters can be obtained by analyzing the affinity matrix of graph.

If $v_{i}$ and $v_{j}$ belong to the same class, then $w_{ij}=1$, otherwise $w_{ij}=0$. According to perturbation analysis of spectral clustering [27], a permutation matrix P always exists to make elements of any node set V in the sequence belongs to a class after the PV transformation. The affinity matrix W is a block diagonal matrix that consists of $n_{k}$ all-1 matrices. Thus, the elements of Laplacian matrix ${\hat{L}}_{sym}$ is divided into *k* matrix blocks. For each matrix block ${\hat{L}}_{n_{i}}$, $\lambda_{k}=n_{i}$, $k=1,2,\ldots n_{i}-1$, where $\lambda_{k}$ is the eigenvalues of matrix block ${\hat{L}}_{n_{i}}$, and $\lambda_{n_{i}}=0$. In light of Matrix Theory [28], the union of the eigenvalues of real symmetric matrix equals that of the block diagonal matrix that consists of these real symmetric matrices. Therefore, the eigenvalues of Laplacian matrix is the union of the eigenvalues of *k* matrices. The eigenvalues of Laplacian matrix consists of $\left(n-k\right)\left(n_{i}-1\right)$ nonzero eigenvalues and *k* zero eigenvalues. The number of clusters is the number of zero eigenvalues of Laplacian matrix ${\hat{L}}_{sym}$.

### 4.2. k-Short Paths Algorithm

In the cost flow network of multitarget tracking, there may be multiple paths that are directed and paths are edge- and node- disjoint, which means that any two paths cannot share the same edge and node, and a path visits a node in the sub-graph at most once. Here, a path represents a possible track. We reformulated the multitarget tracking problem as an edge- and node- disjoint *k*-shortest paths problem on a directed acyclic graph. In order to obtain the *k*-shortest paths, the segmented graph is transformed into a undirected graph firstly, and then the A* search algorithm is adopted to find the single shortest path. If the maximum iteration count is not reached, remove nodes except source node and sink node on the single shortest path, and search for the next path until reaching the maximum iteration count.

### 4.3. Track Mosaic

To segment an undirected graph in the multitarget tracking environment using the adaptive spectral clustering algorithm, it may arise over segmentation. For example, a trajectory may be divided into several segments. In order to obtain the integral track, the mosaic technique is employed to deal with these segmented trajectories. Let $T_{i}$ and $T_{j}$ are two trajectories of different sub-graphs. $x_{io}$ and $x_{jo}$ are the initial position, and $x_{id}$ and $x_{jd}$ are the terminal position, respectively. The Euclidean distance $d_{D}$ is used to decide whether to mosaic two trajectories. If $d_{D}<\tau$, $T_{i}$ and $T_{j}$ are mosaicked, where τ is the mosaic threshold.

### 4.4. Rauch–Tung–Striebel Smoother

A Rauch–Tung–Striebel (RTS) smoother [29] is used to smooth the extracted tracks. The RTS smoother consists of two parts. The first part is the Kalman filter, which calculates the state of target at each time and estimates the corresponding covariance matrix. The second part is backward recursion [30]. In this process, target state and the covariance matrix are taken as inputs to obtain the smoother output.

### 4.5. Time Complexity

The proposed algorithm is parallel processing in that each sub-graph uses the A* search algorithm to obtain the shortest path. The time complexity of the proposed algorithm is mainly composed of two parts, which are the time complexity of the adaptive spectral clustering algorithm and the worst time complexity of searching sub-graph. The time complexity of the adaptive spectral clustering algorithm is $O\left(n\times \left(N-n\right)\right)+O\left(n^{3}\right)+O\left(KNI\right)$. O(n×(N−n)), $O(n^{3})$ and O(KNI) are the time complexity of calculating degree $\hat{d}$, orthogonal eigenvectors $U_{v}$ and K-means clustering algorithm, respectively. *N* is the number of nodes in the undirected graph, *n* is the sample points, *K* is the number of trajectories and *I* is the iteration number of K-means algorithm. The worst time complexity of searching sub-graph is $O(n_{\max}^{2})$, and that $n_{\max}$ is less than *N*. The proposed algorithm is adopted to calculate the *k*-shortest path in $O\left(n\times \left(N-n\right)\right)+O\left(n^{3}\right)+O\left(KNI\right)$. The A* search algorithm, SPFA and SSP are recognized as three effective methods to solve the *k*-shortest path problem. The time complexity of these algorithms are $O(N^{2})$, O(NE) and $O(KN^{2})$, respectively. *E* is the number of edge in the undirected graph. The complexity of these algorithms are primarily related to the value of *K* and *N*. For multitarget tracking systems, that is, *N* is large, and the time complexity of the proposed algorithm is far less than that of the algorithms mentioned above.

## 5. Experimental Results

In this section, the proposed algorithm was tested in different tracking scenarios. The optimal subpattern assignment (OSPA) [31] metric is used for performance assessment. The experiments have been performed on a computer with an Intel G840 2.8 GHz CPU and 4 GB of memory.

### 5.1. Clustering Quality Evaluation

To evaluate the clustering quality of the adaptive spectral clustering algorithm, the external quality measure (F-score) [32] and (MCR) [33] are used. F-score is used in spam detection for documentation as an overall assessment performance that combines the precision and recall ideas from information retrieval. F-score is defined as follows:

(24) $$F-\mathrm{score}\;=\sum_{i=1}^{K}(N_{i}\times \frac{2P_{f}\left(i,j\right)R_{f}\left(i,j\right)}{P_{f}\left(i,j\right)+R_{f}\left(i,j\right)})/N,$$

where $P_{f}\left(i,j\right)=N_{ij}/N_{j}$ represents the precision of the cluster *j* for the given class *i*. $R_{f}\left(i,j\right)=N_{ij}/N_{i}$ represents the recall of the cluster *j* for the given class *i*. $N_{i}$ is the number of the members of class *i*. $N_{ij}$ is the number of numbers of class *i* in cluster *j*. The MCR is given by

(25) $$MCR=\frac{C_{m}}{C_{t}},$$

where $C_{m}$ is the number of misclassified targets. $C_{t}$ is the total number of targets.

**Figure 3:**
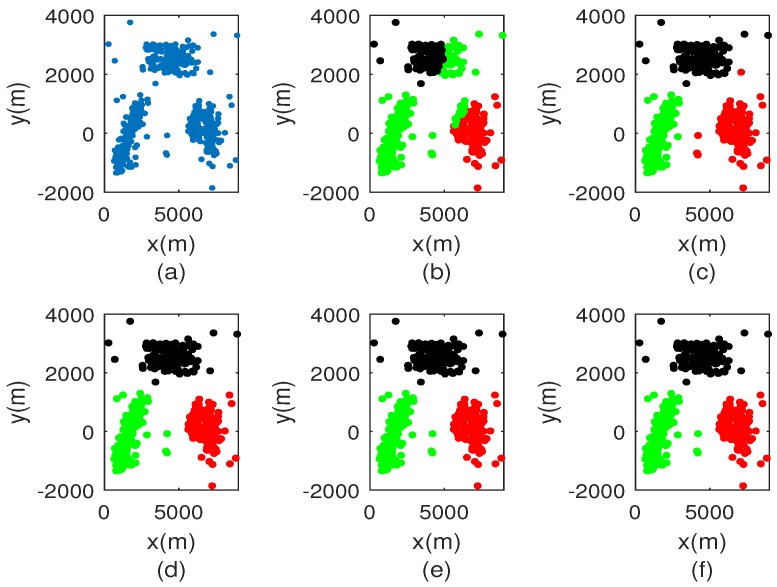
Comparison of classification results. (**a**) original data set; (**b**) sampling rate is 1%; (**c**) sampling rate is 10%; (**d**) sampling rate is 50%; (**e**) sampling rate is 70%; (**f**) sampling rate is 100%.

**Figure 4:**
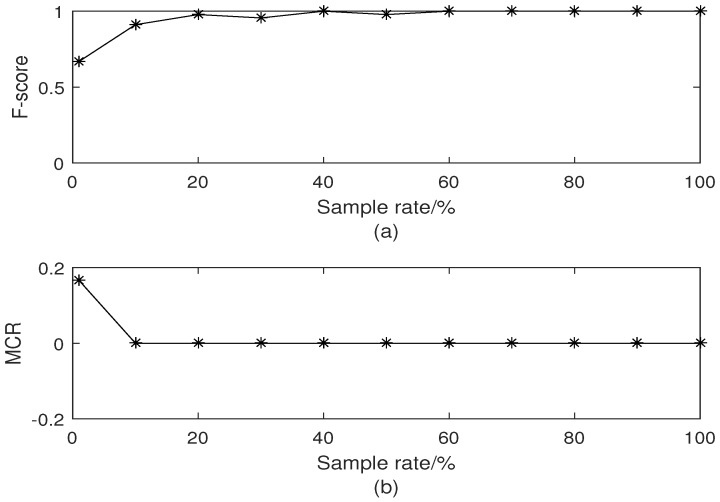
Clustering performance versus sampling rate. (**a**) F-score versus sampling rate; (**b**) MCR versus sampling rate.

Figure 3 displays the comparison of classification results with different sampling rates. Since the sampling rate is 1%, clustering result is seriously affected. When the sampling rate exceeds 1%, it is clear that the classification results outperform that shown in Figure 3b. The clustering performance versus sampling rate are shown in Figure 4. It can be noted that F-score increases with increasing sample rate. Once the sample rate exceeds 30%, the data set can be accurately segmented. This is because the similarity matrix between the sampling points can be approximated by that of all points. In Figure 4b, the sampling rate is equal to 1%, MCR is 0.18. The MCR of other sampling rate is equal to 0. Based on the two evaluation criteria above, we choose the sampling rate as 10% in this paper.

### 5.2. Performance Analysis

In this subsection, we consider two scenarios for multitarget tracking. There are two types of dense clutter areas. Inside Type I dense clutter area, clutter points are uniformly distributed in the surveillance region. While Type II dense clutter area is elliptical and the position of its clutter points follows a 2D Gaussian distribution, whose mean is target position at *k* time and the standard deviations are determined by the major axes of the ellipse. The measurements are obtained from radar which located at [0, 0] m. The measurement model is described as

(26) $$z_{t}^{i}=H\Phi_{t}^{i}+v_{t}^{i},$$

where $\Phi_{t}^{i}={\left[\phi_{t}^{i},{\dot{\phi }}_{t}^{i},\varphi_{t}^{i},{\dot{\varphi }}_{t}^{i}\right]}^{T}$ is the state variable. The measurement noise $v_{t}^{i}$ is zero-mean Gaussian random vector with covariance matrix $R=diag(\left[\delta^{2},\delta^{2}\right])$, and δ=10 m in the scenarios 1 and 2.

The target motion can be modeled by combination of constant turn rate (CT) motion and constant velocity (CV) motion [34]. The dynamistic model of the target is described as follows:

(27) $$\Phi_{t}^{i}=F_{t}^{i}\Phi_{t-1}^{i}+w_{t}^{i},$$

where $F_{t}^{i}$ is the state transition matrix of the target *i* at the time *t*. Under the assumption of CV motion, it is defined as follows:

(28) $$F_{t}^{i}=\left[\begin{array}{cccc} 1 & T & 0 & 0\\ 0 & 1 & 0 & 0\\ 0 & 0 & 1 & T\\ 0 & 0 & 0 & 1 \end{array}\right],$$

where *T* denotes the sampling time. In CT motion, it is defined as

(29) $$F_{t}^{i}=\left[\begin{array}{cccc} 1 & \frac{\sin(\omega_{t}^{i}T)}{\omega_{t}^{i}} & 0 & \frac{\cos(\omega_{t}^{i}T)-1}{\omega_{t}^{i}}\\ 0 & \cos(\omega_{t}^{i}T) & 0 & -\sin(\omega_{t}^{i}T)\\ 0 & \frac{1-\cos(\omega_{t}^{i}T)}{\omega_{t}^{i}} & 1 & \frac{\sin(\omega_{t}^{i}T)}{\omega_{t}^{i}}\\ 0 & \sin(\omega_{t}^{i}T) & 0 & \cos(\omega_{t}^{i}T) \end{array}\right],$$

where $\omega_{t}^{i}$ denotes the turn rate. The process noise $w_{t}^{i}$ is zero-mean Gaussian random vector with covariance matrix

(30) $$Q=\left[\begin{array}{cccc} T^{3}/3 & T^{2}/2 & 0 & 0\\ T^{2}/2 & T & 0 & 0\\ 0 & 0 & T^{3}/3 & T^{2}/2\\ 0 & 0 & T^{2}/2 & T \end{array}\right].$$

Scenario 1:

A multiple non-crossing tracking scenario is considered in the surveillance region [0, 9000] m × [−2000, 5000] m. There are four manoeuvring targets whose initial position are [8000, 2500] m, [500, 3000] m, [2500, 2000] m and [2500, −1500] m, respectively. Their initial velocities are [−80, −80] m/s, [50, −130] m/s, [0, 280] m/s and [150, 0] m/s, respectively. The initial and terminal time are [1, 1, 3, 1] s and [27, 30, 30, 28] s, respectively. The mosaic threshold τ=10.

In this case, the Type I clutter intensity is $\kappa_{1}\left(z_{c}\right)=\frac{m_{Ic}}{V_{I}}=\frac{5}{6.3\times 10^{7}}=0.79\times 10^{-7}$, where $m_{Ic}$ is the expected number of clutter measurements in this Type I dense clutter area, and $V_{I}$ is the surveillance region. For the Type II dense clutter, the expected number of clutter measurements in this Type II dense clutter area is 10, and $V_{II}=4.54\times 10^{5}$ m${}^{2}$ is the ‘volume’ of the Type II dense clutter surveillance region. Therefore, the Type II clutter intensity is $\kappa_{2}\left(z_{c}\right)=\frac{m_{IIc}}{V_{II}}=\frac{10}{\pi \times 600\times 400}=1.33\times 10^{-5}$. We perform a total of 100 Monte-Carlo runs to obtain the average optimal subpattern assignment (OSPA) distance [35] and average of the estimated number of targets.

**Figure 5:**
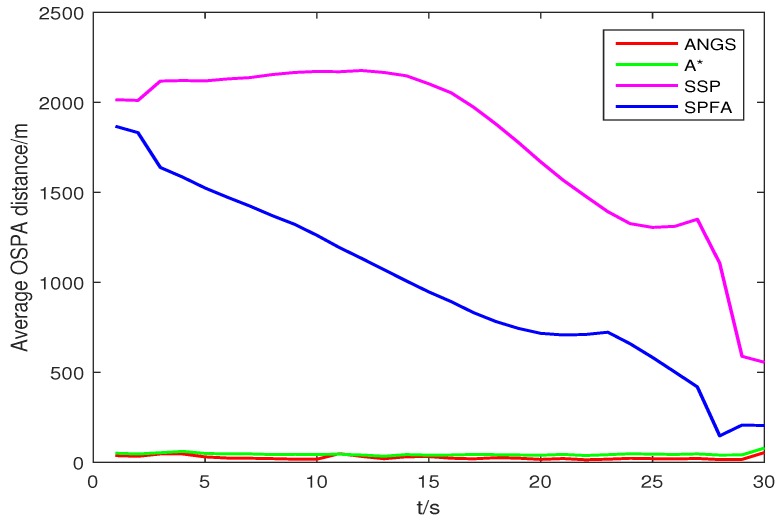
The average OSPA distance in scenario 1, with *c* = 10 and *p* = 2.

**Figure 6:**
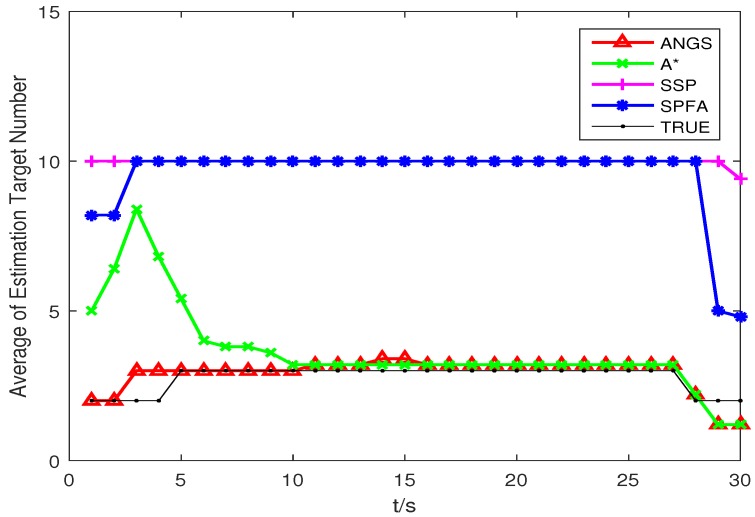
The average of the estimated numbers of targets in scenario 1.

**Figure 7:**
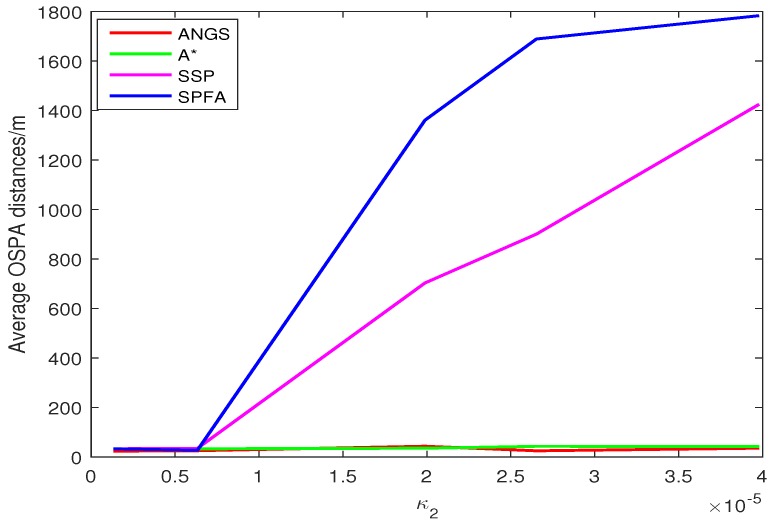
The average OSPA distances versus the Type II clutter intensity in scenario 1.

Figure 5 shows the average OSPA distances of the proposed algorithm, the A* search algorithm, SSP and SPFA. It is observed that the average OSPA distance of the proposed algorithm is approximately equal to that of the A* search algorithm, which are better than that of SPFA and SSP. Figure 6 shows an average of the estimated number of targets of the proposed algorithm, the A* search algorithm, SSP and SPFA. It can be seen that the proposed algorithm presents an considerable performance in the estimation of the target numbers. To make a comparison, set the Type II clutter intensity $\kappa_{2}\left(z_{c}\right)=1.33\times 10^{-6},\;6.33\times 10^{-6},\;19.89\times 10^{-6},\;26.53\times 10^{-6},\;39.79\times 10^{-6}$, respectively. The average OSPA distances of the proposed algorithm, the A* search algorithm, SSP and SPFA versus the Type II clutter intensity are shown in Figure 7.

The average OSPA distances of the proposed algorithm have a relatively small difference than that of other algorithms at $\kappa_{2}\left(z_{c}\right)=1.33\times 10^{-6}$. The average OSPA distance of other algorithms increase rapidly with increasing the clutter intensity. The average OSPA distance of the proposed algorithm is basically no significant change, still at a lower value. When $\kappa_{2}\left(z_{c}\right)=39.79\times 10^{-6}$, it is obvious that the average OSPA distances of SSP and SPFA is about 40 and 50 times of that of the proposed algorithm, respectively.

Scenario 2:

An unknown and time-varying multiple crossing targets scenario is considered in the surveillance region [0, 10,000] m × [−4000, 5000] m. There are four manoeuvring targets whose initial positions are [7000, 4500] m, [3500, 4000] m, [1000, −500] m and [7000, −2500] m, respectively. Their initial velocities are [0, −150] m/s, [200, −20] m/s, [150, 300] m/s and [150, 300] m/s, respectively. The initial and terminal time are [1, 5, 1, 7] s and [27, 30, 30, 27] s, respectively. The mosaic threshold τ=10.

**Figure 8:**
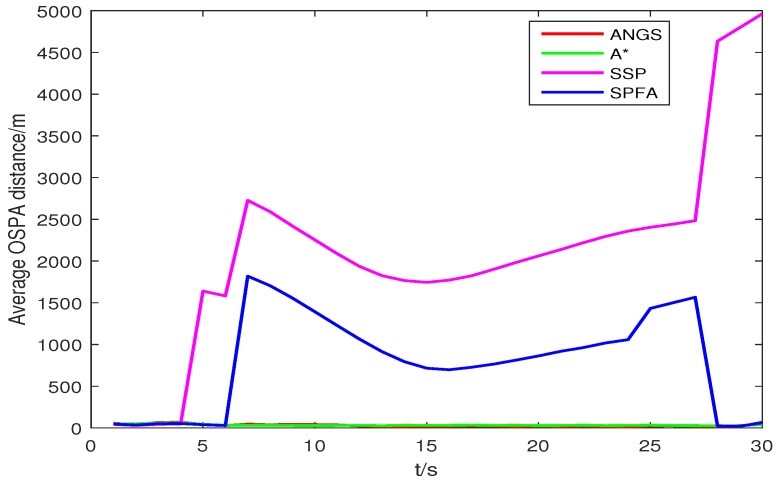
The average OSPA distance in scenario 2, with *c* = 10 and *p* = 2.

**Figure 9:**
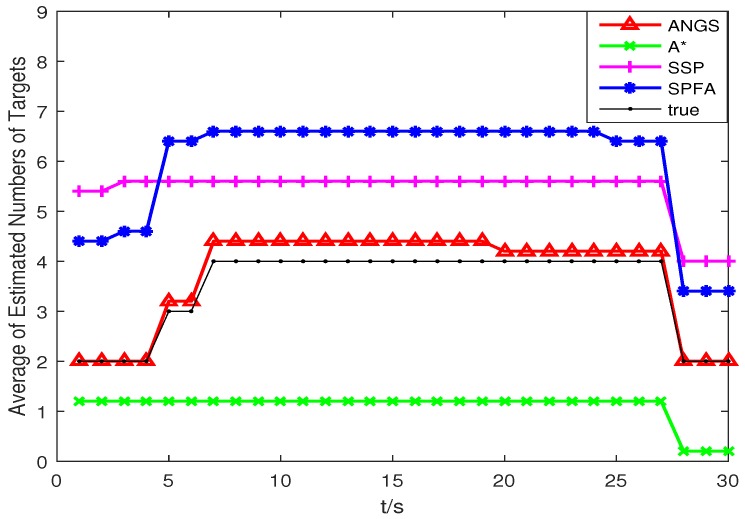
The average of the estimated numbers of targets in scenario 2.

**Figure 10:**
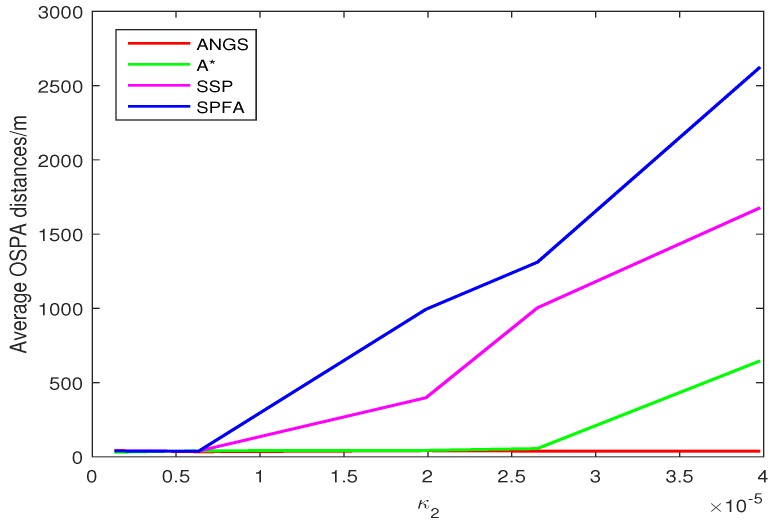
The average OSPA distances versus the Type II clutter intensity in scenario 2.

Trajectories intersect at [7478, 3618] m and [4443, −359] m. The average OSPA distances and the average of the estimated numbers of targets are shown in Figure 8 and Figure 9. The average OSPA distances of the proposed algorithm, the A* search algorithm, SSP and SPFA are given in Figure 10. The Type I clutter intensity is $\kappa_{1}\left(z_{c}\right)=\frac{5}{9\times 10^{7}}=0.56\times 10^{-7}$, and the Type II clutter intensity are $\kappa_{2}\left(z_{c}\right)=1.33\times 10^{-6},\;6.33\times 10^{-6},\;19.89\times 10^{-6},\;gaihao26.53\times 10^{-6},39.79\times 10^{-6}$, respectively.

As seen from Figure 8, the SSP and SPFA have a larger average OSPA distances. In Figure 9, it is apparent that the average target number estimation of the proposed algorithm is exactly the same as the number of the true targets. In Figure 10, as expected, there is an overall increase of OSPA distances with with increasing the clutter intensity. The average OSPA distance of the proposed algorithm is at a lower value. When $\kappa_{2}\left(z_{c}\right)=39.79\times 10^{-6}$, the average OSPA distances of SSP and SPFA is about 45 and 70 times of that of the proposed algorithm, respectively.

### 5.3. Run Time

**Figure 11:**
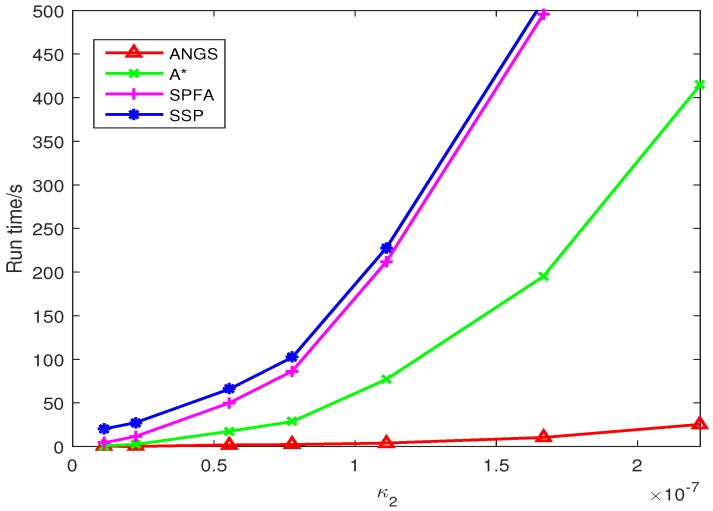
The comparison of average running time.

The average running time of different Type II clutter intensity is shown in Figure 11. It clear that the running time of the A* search algorithm, SSP, and SPFA increase exponentially. The running time of the proposed algorithm is growing slowly. When $\kappa_{2}\left(z_{c}\right)=2.222\times 10^{-7}$, the running time of the A* search algorithm is about 14 times that of the proposed algorithm.

## 6. Conclusions

In this paper, we have presented a novel data association framework for multitarget tracking with measurement uncertainty that estimates unknown number and states of targets using the continuous multi-frame data. The multitarget tracking problem was formulated as network flow optimization problem for finding *k*-shortest paths, and an adaptive spectral clustering algorithm was used to segment the network structure. The optimal solution of each sub-graph can be obtained by the A* search algorithm. Experiment results indicate that the proposed algorithm is helpful in improving the accuracy of track extraction and can reduce the computational complexity. Future work will focus on tracking multitargets with low detection probability.
